# Identification of immuno-infiltrating MAP1A as a prognosis-related biomarker for bladder cancer and its ceRNA network construction

**DOI:** 10.3389/fonc.2022.1016542

**Published:** 2022-11-02

**Authors:** Xiaoyue Lyu, Yujie Qiang, Bo Zhang, Wei Xu, Yali Cui, Le Ma

**Affiliations:** ^1^ College of Life Sciences, Northwest University, Xi’an, China; ^2^ Ankang Hospital of Traditional Chinese Medicine, Ankang, Shaanxi, China

**Keywords:** MAP1A, bladder cancer, diagnosis, prognosis, immuno-infiltrating, ceRNA network

## Abstract

**Backgrounds:**

Approximately 75% of bladder cancer occurrences are of the non-muscle-invasive type. The estimated five-year survival rate is 26%–55%. Currently, there is no reliable biomarker available for early diagnosis and prognosis of bladder cancer. The present study aims to identify a biomarker using bioinformatic approaches to provide a new insight in clinical research for early diagnosis and prognosis of bladder cancer.

**Methods:**

Clinical data and a transcriptome of bladder cancer were obtained from TCGA, GEO, GETx, and UCSC Xena. The differential expressed gene (DEG) analysis, weighted gene co-expression network analysis (WGCNA), and survival analysis using the Kaplan-Meier and Cox proportional-hazards models were used to identify the Microtubule-associated Proteins 1A (MAP1A). on overall survival (OS) and disease-free survival (DFS) was analyzed using GEPIA and GETx databases. The TIMER 2.0 database predicted the correlation between MAP1A and immunocytes and immune checkpoints. Target prediction of the regulated competing endogenous RNAs (ceRNAs) network of MAP1A was performed using starBase and TargetScan. Cystoscope v3.7.2 software was used to visualize the ceRNA coexpression network. The R programming language v4.0.2 was applied as an analytic tool. Gene expression of MAP1A verified by RT-qPCR.

**Results:**

The low expression of MAP1A was verified in bladder cancer tissues and bladder cancer cell lines SW780 and 5637. P < 0.001 were obtained by Kaplan-Meier survival analysis and Cox proportional hazards model, with a hazard ratio (HR) of 1.4. Significant correlations between MAP1A and OS (P < 0.001, HR = 1.9) as well as DFS (P < 0.05, HR = 1.7) in bladder cancer were identified through gene expression profiling interactive analysis (GEPIA), indicating MAP1A may be a high-risk factor. Significant correlation in single copy-number variation of MAP1A gene with CD8^+^ T cells, and myeloid dendritic cells (MDCs) (P < 0.05) was noted. MAP1A expression was shown to be significantly correlated with the amount of CD4^+^ T cells and CD8^+^ T cells, MDCs, macrophages, and neutrophils in a statistically significant positive manner (P < 0.001). However, the MAP1A expression demonstrated a strong negative connection with B cells (P < 0.001). Except for macrophage M1 genes IRF5 and PTGS2, MAP1A expression was significantly correlated with the gene levels in immunocytes such as CD4^+^ T cells, CD8^+^ T cells, B cells, dendritic cells (DCs), macrophages, and neutrophils (Cor > 0.2, P < 0.001), as well as immune checkpoint related genes including cytotoxic t-lymphocyte-associated protein 4 (CTLA-4), programmed death 1 (PD-1), programmed death ligand 1 (PD-L1) (P < 0.001). Finally, we predicted that the MAP1A-interacting miRNA was miR-34a-5p, and the MAP1A endogenous competing RNAs were LNC00667, circ_MAP1B, and circ_MYLK, respectively. These findings support the need for further studies on the mechanism underlying the pathogenesis of this disease.

**Conclusion:**

MAP1A is considered as a prospective biomarker for early diagnosis, therapeutic observation, and prognosis analysis in bladder cancer.

## Introduction

Bladder cancer (BC) is one of the world’s top 10 common cancer diseases. According to GLOBOCAN, nearly 0.57 million are diagnosed of bladder cancer worldwide with 0.21 million deaths in 2020. There is an increase in both newly diagnosed cases and overall death from 2018 (0.55 million and 0.20 million) to 2020. The morbidity and mortality of bladder cancer rank 6^th^ and 9^th^ respectively among the 36 common cancers in men for the year 2020 (1, 2). According to the National Comprehensive Cancer Network (NCCN) version 4. 2020 bladder cancer, over 90% of bladder cancers are urothelial cancers, accounting for more than 75% of non-muscle invasive bladder cancers (3). However, clinically, 91% of non-muscle invasive bladder cancers are concurrent with 2 to 3 superficial muscle-invasive balder cancers, (≤ T2a, ≤ Grade II, according to American Joint Committee on Cancer Staging (AJCC) cancer staging and World Health Organization cancer grading), with a 5-year survival rate of 77.1%, a 5-year relapse rate of 31%~78% and a 5-year progression rate of over 45% (3–8).

The Microtubule-associated Proteins 1A (MAP1A), is expressed mainly in neurons, and contributes to the development of microtubules, axons, and dendrites. This gene encodes a pro polypeptide, which is further hydrolyzed to form the heavy chain and light chain of MAP1A. A mature complex MAP1A-LC2/LC3 is produced through protein assembly with light chain (LC)2/3, a separate encoding subunit. This complex plays an important role in disease development. The gene expression of MAP1A is almost exclusively restricted to the brain (9–12). Currently, few studies have reported the role of MAP1A in disease but have focused on MAP1A targeting therapy for nervous system diseases, including depression and Parkinson’s disease, the correlation between MAP1A gene expression and toxicity of HIV-1 infection-related immune diseases, as well as the degree of malignancy in brain glioma and prostate cancer (13–17).

**Figure 1:**
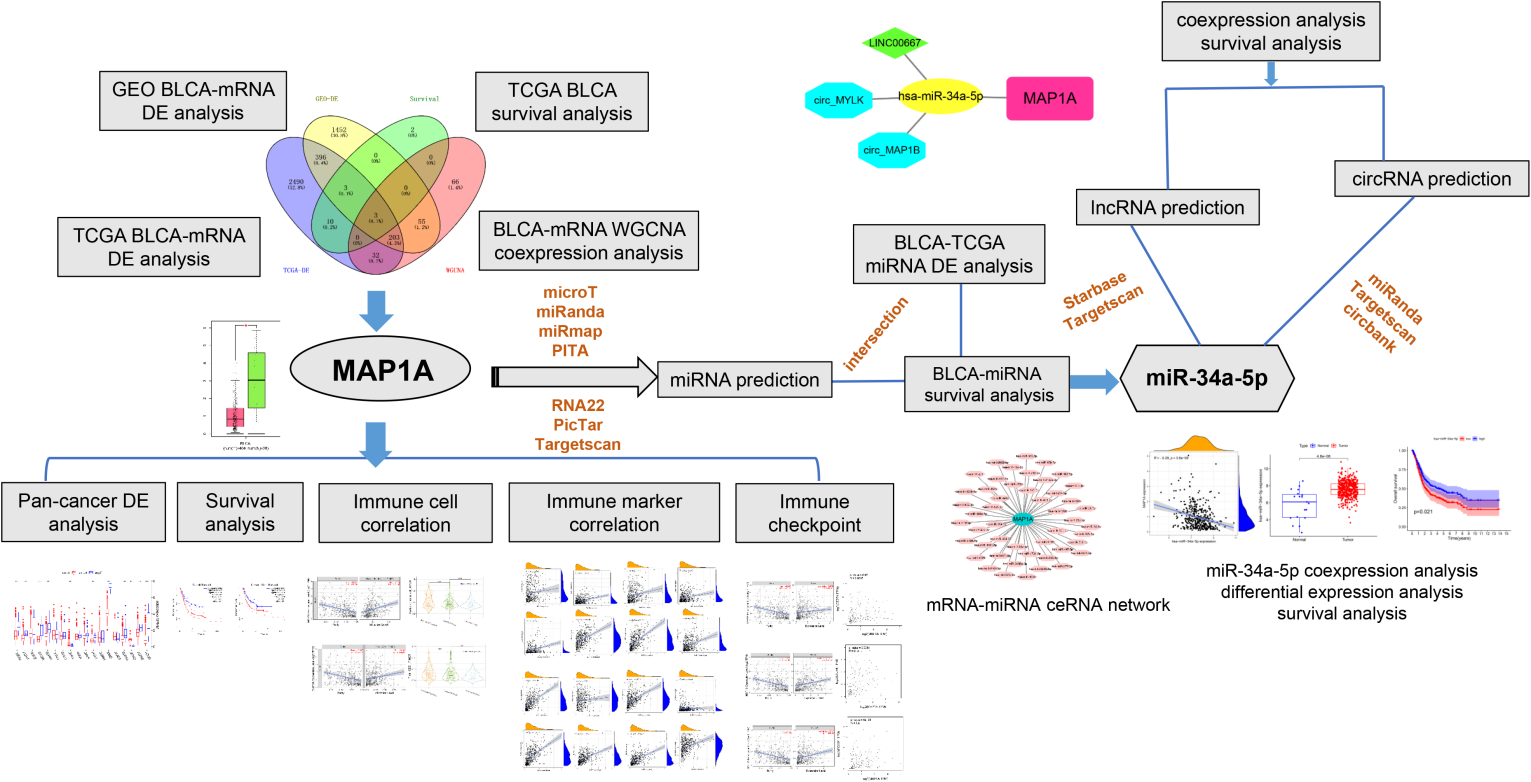
Workfolw. We first acquired bladder cancer transcriptome data from the TCGA and GEO databases, and then we performed differential expression, WGCNA, and survival analysis to focus on the research target MAP1A. Then, for the MAP1A gene, we performed pan-cancer analysis, correlation analysis with immune cells and immunological checkpoints, and confirmed the MAP1A expression and survival analysis in the GEPIA database. The expression of MAP1A was validated in bladder cancer cell lines. Finally, the mechanism of the MAP1A endogenous competitive RNA network was hypothesized.

This study assessed the gene MAP1A expression in bladder cancer and correlated to the survival bioinformatically from TCGA and GEO databases and verified by differential gene expression and survival analysis using the GEPIA database. Further, *in vitro* confirmation of MAP1A expression at the cellular level was done using bladder cancer cell lines SW780 and 5637, as well as normal bladder epithelial cells SV-HUC-1. Additionally, the R language was used to analyze the expression of MAP1A in pan-cancer. The TMIER2.0 database and R language were utilized to assess the correlation between MAP1A and immune cells, immune cell-related genes, as well as immunological checkpoint genes. Databases like Starbase and TargetScan were applied to predict the mRNA-miRNA-lncRNA/circRNA endogenous competitive RNA interaction network interactions. The workflow is presented in **Figure 1**. The present work reports for the first time that the gene MAP1A could be a potential biomarker in the early diagnosis, prognosis, and immunotherapy of low-grade bladder cancer (non-muscle invasion and superficial muscle invasion stages).

## Methods

### Data source and processing

TGCA-BLCA transcriptomes and clinical data (survival, time of follow-up, staging and level, gender, age) were collected from The Cancer Genome Atlas database (https://portal.gdc.cancer.gov/). A total of 151 samples were used in the current study, including 132 bladder cancer samples (T ≤ II, Grade ≤ IIa, N0M0) and 19 normal samples. Dataset GSE19915 was obtained from National Center for Biotechnology Information-Gene Expression Omnibus (NCBI-GEO) database (https://www.ncbi.nlm.nih.gov/geo/) (18), which contained 91 bladder cancer samples and 7 normal tissue samples. Pan-cancer gene expression and survival data were downloaded from the the University of California, Santa Cruz (UCSC) Xena database (https://xena.ucsc.edu/) (19). The edgeR package (20) in R was applied to standardize data and analyze differential expression (P < 0.01, |logFC| ≥ 2), and the Practical Extraction and Report Language (Perl) was used to filter and combine data (21) to make a more accurate analysis of the data from TCGA-BLCA-RNA-Seq and NCBI-GEO-GSE19915.

### Survival analysis

Survival TCGA-BLCA was downloaded from the TCGA database. Key information, including time of survival and follow-up, gender, age, and sample number, was then extracted. Limma (22) and survival (23) packages in R were used to identify genes associated with prognosis (P < 0.001 was defined to obtain enormously significant correlated genes). The Product-Limit method (i.e., the Kaplan-Meier method) (24) and the Cox model (25) were combined to screen genes for correlation to survival.

### Weighted gene co-expression analysis

The WGCNA package in R (26) was applied to perform module identification and the dividing of transcriptomes from TCGA-BLCA and GEO-GSE19915 according to correlation with bladder cancer. A topological overlap matrix (TOM) was used to measure the connection strength between genes (Dynamic Tree Cut: minModuleSize = 50; Cluster Cut: MEDissThres = 0.25). Candidate modules were defined as significantly correlated with clinical characteristics, i.e. significant differential gene expression between normal tissue and bladder cancer tissue.

### Pan-cancer analysis

In this study, the transcriptomes of 33 different cancer types were obtained from the UCSC Xena database. The limma package in R was used to analyze the expression of the MAP1A gene in pan-cancer tissues.

### Correlation analysis of immunocytes and immune checkpoints

The TIMER database (http://timer.cistrome.org/) (27) was used for correlation analysis of MAP1A copy number and its expression with immunocytes involving CD4^+^ T cells, CD8^+^ T cells, B cells, Dcs, and macrophages. Using both the TIMER database and GEPIA data, the correlation between MAP1A and immune checkpoint (PD-1/PD-L1, CTLA4) specific genes (CD274, PDCD1, CTLA4) volume was investigated in order to obtain more accurate predictions. Correlation between immunocyte genes and the gene MAP1A was analyzed using the limma package in R.

### CeRNA network construction

In order to discover miRNAs that may be regulated by MAP1A (28–30), we used databases such as microT, miRanda, PITA, RNA22, PicTar, and Targetscan. The TCGA-BLCA-miRNA expression profile and clinical data were downloaded from the TCGA database. The limma package in R was used to screen differentially expressed miRNAs in bladder cancer and the survival package for finding miRNAs associated with patient survival. Candidate miRNAs were screened as MAP1A targeted, with a negative correlation with MAP1A expression and associated with survival in bladder cancer. The target lncRNAs of miRNAs were predicted using the Starbase and Targetscan databases (28, 29) and regulated circRNAs were predicted using Targetscan, Circbank, and miRanda (28, 29, 31). The prediction of miRNA-targeted non-coding RNAs (lncRNAs and circRNAs) and their correlation with miRNAs and MAP1A expression in bladder cancer were further analyzed using the limma package in R. Candidate circRNAs were defined based on corFilter = 0.2 and pvalueFilter = 0.001, and lncRNAs were defined based on corFilter = 0.2 and pvalueFilter = 0.05. The correlation (P < 0.001) between non-coding RNAs and survival in bladder cancer patients was analyzed using both the limma and survival packages in R. A ceRNA network of MAP1A-miRNA-lncRNA/circRNA was constructed after the identification of miRNAs negative correlation with MAP1A expression in bladder cancer, as well as lncRNAs and circRNAs positive correlation with MAP1A. Cytoscape v3.7.2 was finally used to visualize the ceRNA network.

### Cell lines and culture

Human bladder carcinoma SW780 (Grade I) and 5637 (Grade II) cells, as well as human bladder epithelial cell SV-HUC-1, were obtained from Procell Life Science & Technology Co., Ltd. (Wuhan, China). All the above cell lines were certified and routinely tested for mycoplasma contamination before use. For SV-HUC-1 cells, the culture medium consisted of Ham’s F-12K supplemented with 10% FBS (GIBCO, USA) and 1% Penicillin/Streptomycin (Invitrogen, USA), while for 5637 cells, RPMI-1640 supplemented with 10% FBS (GIBCO, USA) and 1% Penicillin/Streptomycin (Invitrogen, USA) were used and grew under 95% air and 5% CO2 at 37°C. SW780 cells were cultured in Leibovitz’s L-15 supplemented with 10% FBS (GIBCO, USA) and 1% Penicillin/Streptomycin (Invitrogen, USA) under 100% air at 37°C.

### RNA extraction and RT-qPCR assay

Total RNA was extracted using TRIzol Universal Reagent (TIANGEN, Beijing, China). The reverse transcription and quantification were conducted using the PrimeScript TM RT reagent Kit (Takara, Dalian, China) and the TB Green Premix Ex TaqTM Kit (Takara, Dalian, China), following instructions. The Primer Premier 5 software was used to design the PCR primers and the primer synthesis was performed by Tsingke Biotechnology Co., Ltd. (TSINGKE, Xi’an, China). The RT-qPCR assay was performed on an ABI PRISM 7500 Sequence Detection System (Applied Biosystems, USA). The 2^-△△CT^ method was applied to analyze the relative expression of MAP1A. A comparison between different types of cells was conducted using one-way ANOVA analysis. The statistical difference was set at P < 0.05. The following primer sequences were used in the current study: The forward primer sequence for GAPDH (internal control) is CAATGACCTTCATTGACC, and the reverse primer sequence is TTGATTTTGGAGGGATCTCG; the forward primer sequence for MAP1A is GCCGGCATCAATGGACTAC, and the reverse primer sequence is CAACTCCAAGCTCAAGAGAT.

### Statistical analysis

Differential gene expression was determined according to the Wilcox test and P-value. Pearson’s correlation coefficient was used to analyze the correlation. Survival analysis was determined according to HR and P values. A P value of less than 0.05 was considered statistical significance, less than 0.01 was considered a significant difference, and less than 0.001 was considered an extremely significant difference. An HR value of more than 1.0 indicates high risk.

## Results

### Differential expression analysis

**Figure 2:**
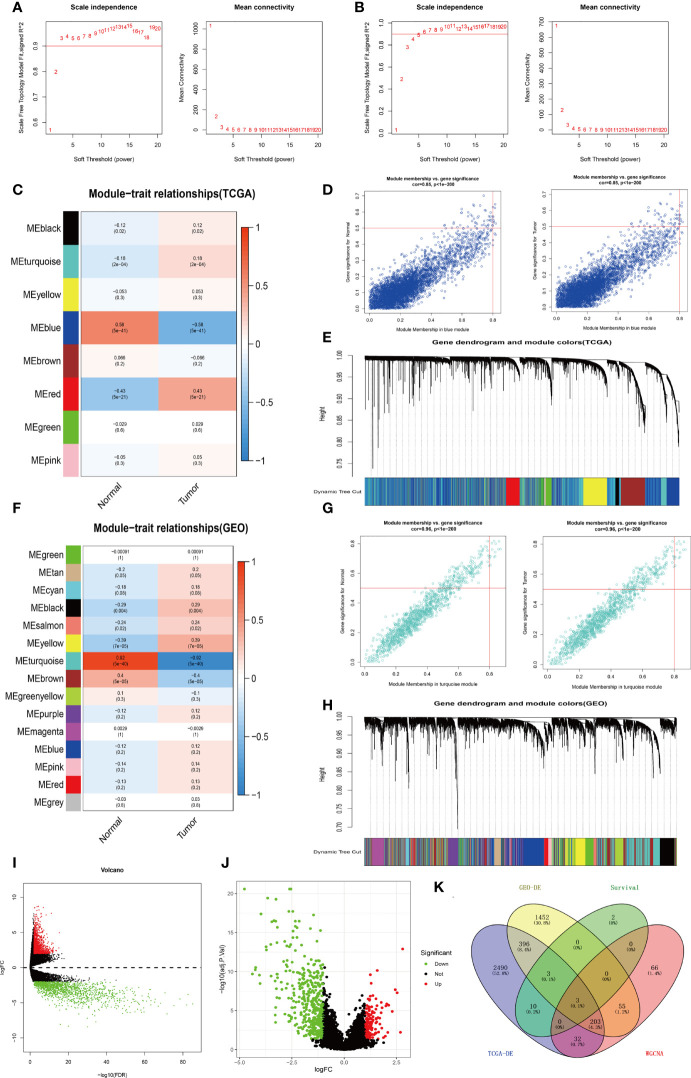
Process of screening for target genes. **(A)** Analysis of network topology for various soft-thresholds (power) in the TCGA dataset. **(B)** Network topology analysis in the GEO dataset for various soft-thresholds (power). **(C)** Heat map of the correlation between the TCGA-WGCNA gene clustering module and clinical traits. **(D)** Correlation of TCGA-WGCNA Blue Modules with clinical traits. **(E)** Modules for each TCGA-WGCNA gene (each branch represents one gene). **(F)** A heat map of GEO-WGCNA gene clustering modules that are associated with clinical traits. **(G)** Correlation of GEO-WGCNA-turquoise modules with clinical traits. **(H)** Modules corresponding to each gene of GEO-WGCNA (each tree branch represents one gene). **(I)** Visualization of TCGA-BLCA-mRNA differential expression using a volcano plot. **(J)** Visualization of GEO-GSE19915-mRNA differential expression analysis volcano map. **(K)** A Venn diagram depicting the TCGA-BLCA differentially expressed genes, GEO-GSE19915 differentially expressed genes, weighted gene co-expression genes, and TCGA-BLCA intersecting genes with survival-related genes.

A total of 2892 differentially expressed genes were obtained using the edgeR package in R (filter: Fold Change = 2, Calibration: P < 0.01) to explore specimens from the TCGA database, including 132 bladder cancer specimens (T ≤ II, Grade ≤ IIa, N0M0) and 19 normal bladder specimens (**Supplementary Material**, **Table 1**). Meanwhile, 2112 differentially expressed genes were obtained through analysis of the GEO-GSE19915 dataset (**Supplementary Material**, **Table2**). Visualized volcano plots are shown in (**Figures 2I, J**).

### WGCNA analysis

The WGCNA package in R was used to analyze TCGA-BLCA transcriptome data. To build a WGCNA network for TCGA and GEO, we first calculated the soft threshold (power), then raised the coexpression similarity to calculate adjacency. We used the pickSoftThreshold function in WGCNA, which performs the analysis of network topology. The soft thresholding (power) was set at 3 and 4 in the TCGA and GEO datasets, respectively. The scale independence reached 0.9 and the average connectivity was relatively high, as shown in **Figures 2A, B**. Eight modules comprising highly correlated genes were identified based on clinical features. The blue module has the most negative correlation with bladder cancer in the present study (P = 5e-41, R = -0.58). Similarly, 15 associated modules with a relationship between clinical features and the GEO dataset were identified. The turquoise module is related to the risk of bladder cancer (P = 5e-40, R = -0.92). Thus, we consider the genes in the blue module from TCGA-WGCNA analysis and the turquoise module from GEO-WGCNA analysis to be the most correlated with bladder cancer in the current research (**Figures 2C–H**).

### Survival analysis

In the present study, the Kaplan-Meier analysis in combination with the Cox regression model was applied to analyze clinical data from TCGA-BLCA. During the screening, the calibration P value was set below 0.001 to obtain the most correlated biomarker. A total of 19 biomarkers were found to correlate with the survival of bladder cancer patients. Among these biomarkers, 11 genes show high risk (HR > 1), including MXRA7, LAMA2, AHNAK, EMP1, MAP1A, PRKG1, SVIL, PID1, NCAM1, JAM3, and EFEMP1 (**Table 1**).

**Table 1:** Results of survival analysis by the KM methodology and the Cox regression approach.

Biomarker	KM (Pvalue)	Cox (Pvalue)	HR
MXRA7	0.000593	1.21E-05	1.468049
LAMA2	0.000649	4.65E-05	1.437145
AHNAK	3.86E-05	1.66E-05	1.409271
EMP1	9.78E-05	2.77E-07	1.35185
MAP1A	3.04E-05	5.40E-05	1.407538
PRKG1	0.000238	0.000742	1.457765
SVIL	0.00011	0.000298	1.326061
PID1	0.00012	0.000685	1.340701
NCAM1	0.000141	0.000787	1.303549
JAM3	0.00049	0.000629	1.279121
EFEMP1	0.000131	0.000143	1.156483

(HR>1, Pvalue<0.001).

### Veen analysis

**Figure 3:**
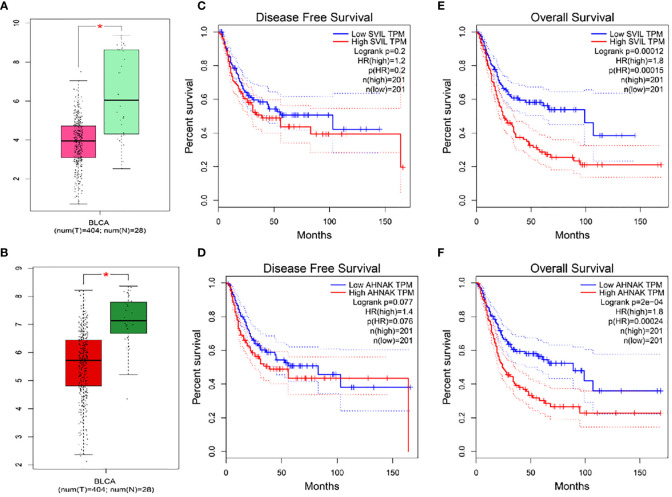
Differential expression analysis and overall survival and disease-free survival of SVIL and AHNAK were validated in the GEPIA database in combination with normal samples in the GETx database. **(A)** SVIL difference expression box visualization diagram; **(B)** AHNAK difference expression box visualization diagram; **(C)** Disease Free Survival for SVIL; **(D)** Disease Free Survival for AHNAK; **(E)** Overall Survival analysis for SVIL; **(F)** Overall Survival analysis for AHNAK. * indicates P < 0.05.

Veen analysis was performed to find common genes using 19 survival-related genes in bladder cancer, 2892 differential expressed genes from TCGA-BLCA, 2112 differential expressed genes from GEO-GSE19915, 4229 genes in the WGCNA-TCGA-blue module, and 1191 genes from the WGCNA-GEO-turquoise module. Three overlap genes, including SVIL, AHNAK, and MAP1A, were finally obtained (**Figure 2K**). To further confirm single genes, normal samples from GEPIA and GETx databases were used to verify differential expression of the above 3 genes, overall survival rate, and disease-free survival rate (**Figure 3**). MAP1A was significantly correlated with overall survival and disease-free survival rates in bladder cancer (P < 0.001). Thus, MAP1A was selected as a target for the following studies.

### Verification of MAP1A gene expression

**Figure 4:**
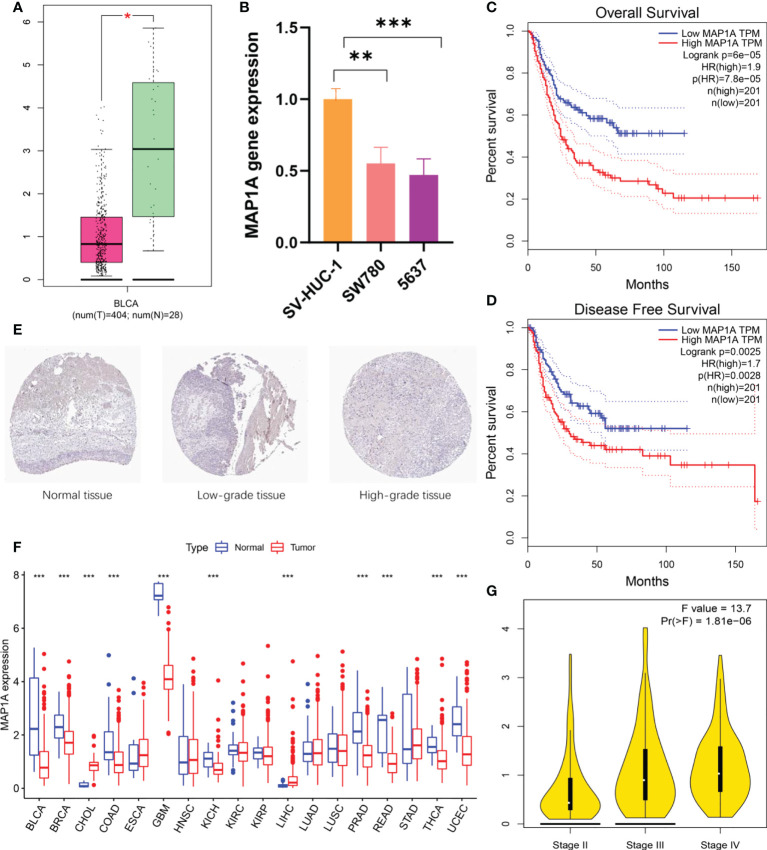
MAP1A gene expression and prognosis validation. **(A)** Validation of MAP1A expression in bladder cancer tissue samples and normal tissue samples using the GEPIA database and the GETx database. **(B)** MAP1A expression in bladder cancer cells SW780 and 5637, as well as normal bladder epithelial cells SV-HUC-1. **(C)** MAP1A in the GEPIA database for overall survival analysis of bladder cancer. **(D)** MAP1A correlation with disease-free survival in bladder cancer (GEPIA database). **(E)** MAP1A expression in bladder cancer tissues (immunohistochemistry, HPA database). **(F)** MAP1A expression in pan-cancer. (* indicates P < 0.05, ** indicates P < 0.01, *** indicates P < 0.001). BRCA, Breast invasive carcinoma; CHOL, Cholangiocarcinoma; COAD, Colon adenocarcinoma; ESCA, Esophageal carcinoma; GBM, Glioblastoma multiforme; HNSC, Head and neck cancer; KIRC, Kidney renal clear cell carcinoma; KIRP, Kidney renal papillary cell carcinoma; LIHC, Liver hepatocellular carcinoma; LUAD, Lung adenocarcinoma; LUSC, Lung squamous cell carcinoma; PRAD: Prostate adenocarcinoma; READ, Rectum adenocarcinoma; STAD, Stomach adenocarcinoma; THCA, Thyroid carcinoma; UCEC, Uterine corpus endometrial carcinoma. **(G)** The expression of MAP1A in different stage of bladder cancer.

**Figure 5:**
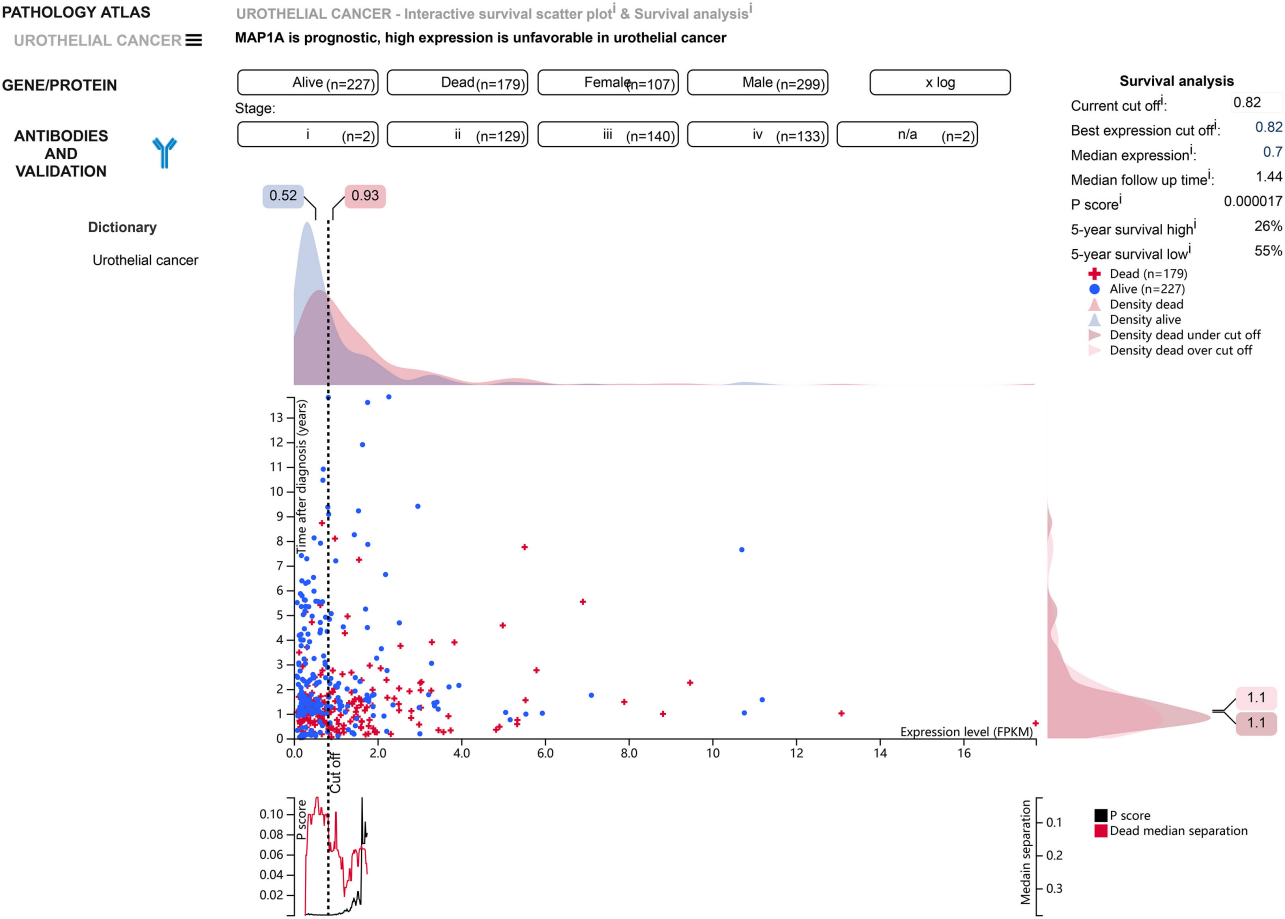
According to the HPA database, MAP1A is associated with a poor prognosis in bladder cancer (P < 0.001), and 5-year survival in bladder cancer patients ranges from 26% to 55%.

Normal bladder samples from GEPIA and GETx databases were used to verify MAP1A gene expression in comparison to bladder cancer tissues. As shown in **Figure 4A**, the MAP1A gene was decreased in bladder cancer tissues compared with high levels in normal bladder samples. The differential expression of MAP1A was statistically significant (P < 0.05). The immunohistochemical observation in Human Protein Atlas (HPA) database demonstrated moderate expression of MAP1A in normal bladder tissues (HPA039064, Patients id: 751, Male, age: 55, Intensity: Moderate, Quantity < 25%), with a weak expression intensity in low-grade bladder cancer (HPA039064, Patients id: 4936, Male, age: 76, Intensity: Weak, Quantity < 25%) and undetectable in high-grade bladder cancer (HPA039064, Patients id:1984, Male, age61, Intensity: Negative, Quantity: None) (**Figure 4E**). Additionally, MAP1A is correlated with a poor prognosis of bladder cancer (P < 0.001), with an approximately 26% ~ 55% of 5-year survival rate (**Figure 5**).

*In vitro*, bladder cancer cell lines (SW780, 5637) and normal bladder epithelial cell SV-HUC-1 were verified for MAP1A expression (**Figure 4B**). MAP1A was highly expressed in SV-HUC-1 cells, whereas the levels were low in both SW780 and 5637 cell lines. Compared to MAP1A in SV-HUC-1, significant differences were found in SW780 (P < 0.01) and 5637 (P < 0.001). (**Supplementary material, Data sheet 1**)

Next, the limma package in R predicted MAP1A expression in pan-cancer (**Figure 4F**). Compared with normal bladder tissues, single-gene MAP1A decreased in various cancers (BLCA, BRCA, COAD, GBM, KICH, PRAD, READ, THCA, and UCEC, P < 0.001), while it increased in CHOL and LIHC (P < 0.001). No significant difference was observed in ESCA, HNSC, KIRC, KIRP, LUAD, LUSC, and STAD. Furthermore, the correlation between MAP1A expression and overall survival as well as disease-free survival was verified using the GEPIA database (**Figures 4C, D**). High expression of MAP1A showed a strong correlation with the overall survival factor (P = 7.8e-05, HR = 1.9), with statistical significance with the disease-free survival risk factor (P = 0.0028, HR = 1.7). Moreover, together with GEPIA analysis, MPA1A showed significant differences within different stages of bladder cancer (F value = 13.7, Pr (>F) = 1.81e-06) (**Figure 4G**).

### Correlation analysis between MAP1A and immunocytes and immune checkpoints

**Figure 6:**
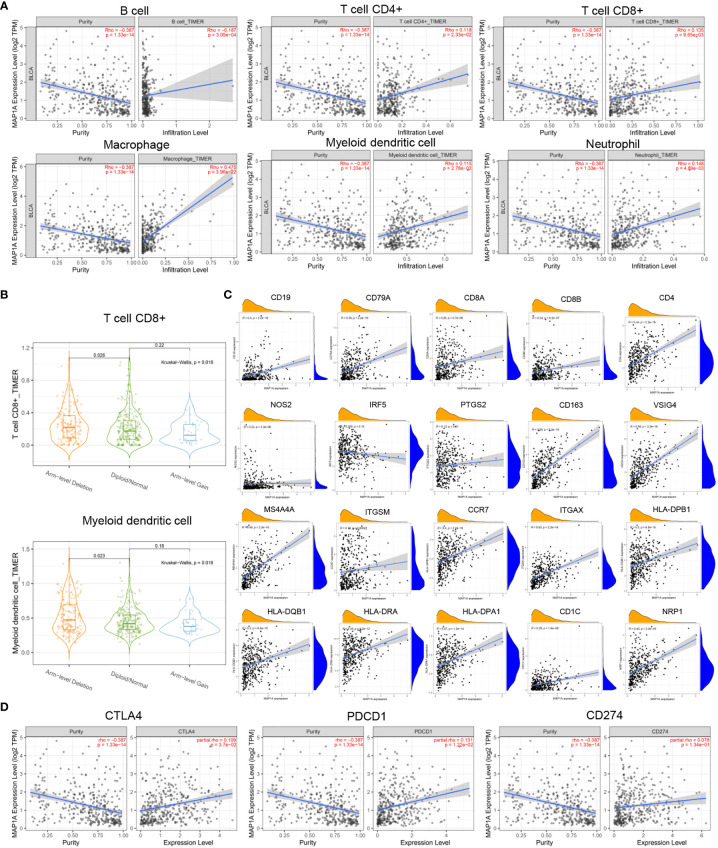
Correlation between MAP1A and immune cells, immune cell genes, and immune checkpoints. **(A)** Visual analysis of the correlation between MAP1A gene expression and the content of immune cells (B cells, T cells, macrophages, DCs, neutrophils). **(B)** Correlation between MAP1A gene copy number and immune cells CD8^+^T cells, mDCs. **(C)** Correlation between B cell genes (CD19, CD79A), CD8^+^T cell genes (CD8A, CD8B), CD4^+^T cell genes (CD4), M1 macrophage genes (NOS2, IRF5, PTGS2), M2 macrophage genes (CD163, VSIG4, MS4A4A), neutrophil genes (ITGAM, CCR7), DC genes (HLA-DPB1, HLA-DQB1, HLA-DRA, HLA-DPA1, CD1C, NRP1, ITGAX), and other immune cell genes expression and MAP1A gene expression were visualized in the correlation analysis. **(D)** A visual analysis of the relationship between the expression of immune checkpoint genes CD274, PDCD1, CTLA4, and MAP1A.

To predict whether MAP1A is correlated with immunocytes, the TIMER2.0 database was used to investigate the correlation between immunocytes and MAP1A copy number variation and its expression. Tumor purity was normalized before correlation analysis. The levels of CD4^+^ T cells, CD8^+^ T cells, mDCs, macrophages, and neutrophils showed a significant positive correlation with single gene MAP1A expression (P < 0.001), with a significant negative correlation for B cells (P < 0.001) (**Figure 6A**).

**Figure 7:**
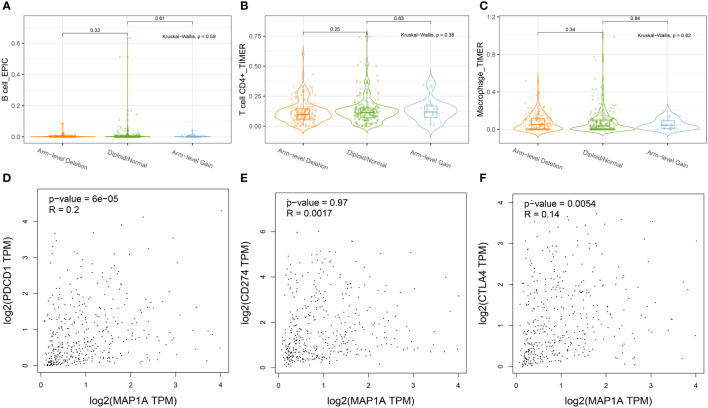
Correlation of MAP1A arm-level deletion with immune cells: **(A)** B cell; **(B)** CD4+ T cell; **(C)** Macrophage. MAP1A correlation analysis with immune checkpoint genes was analyzed by the database GEPIA: **(D)** PDCD1; **(E)** CD274; **(F)** CTLA4.

As shown in **Figure 6B**, the copy number alteration of MAP1A was associated with CD8^+^ T cells and mDCs (P < 0.05). No correlation was noted with B cells, CD8^+^ T cells, macrophages, and neutrophils (**Figures 7A–C**). The limma package in R was applied to analyze the correlation between MAP1A and genes in immunocytes. As displayed in **Figure 6C**, positive correlations (P < 0.01) with MAP1A occurred for genes in B cells (CD19, CD79A), CD8^+^ T cells (CD8A, CD8B), CD4^+^ T cells (CD4), M1 macrophages (NOS2, PTGS2), M2 macrophages (CD163, VSIG4, MS4A4A), neutrophils (ITGAM, CCR7), and DCs (HLA-DPB1, HLA-DQB1, HLA-DAR, HLA-DPA1, CD1C, NRP1, ITGAX). No significant correlation was observed with the IRF5 gene in M1 macrophages (P = 0.13).

To further confirm whether single-gene MAP1A is a potential monitoring biomarker for immunotherapy in bladder cancer, TIMER2.0 and GEPIA databases were used for correlation analysis of immune checkpoint PDL-1/PD1, CTLA4 related gene CD274, PDCD1, and CTLA4. As seen in **Figure 6D**, prediction based on the two databases demonstrated an extremely significant positive correlation of PDCD1 and CTLA4 with MAP1A expression (P < 0.001). However, a significant positive correlation of CD274 with MAP1A was only obtained in the TIMER 2.0 database (P < 0.001), with no correlation in the GEPIA database (P > 0.05) (**Figures 7D–F**).

### Construction of ceRNA network for MAP1A

**Figure 8:**
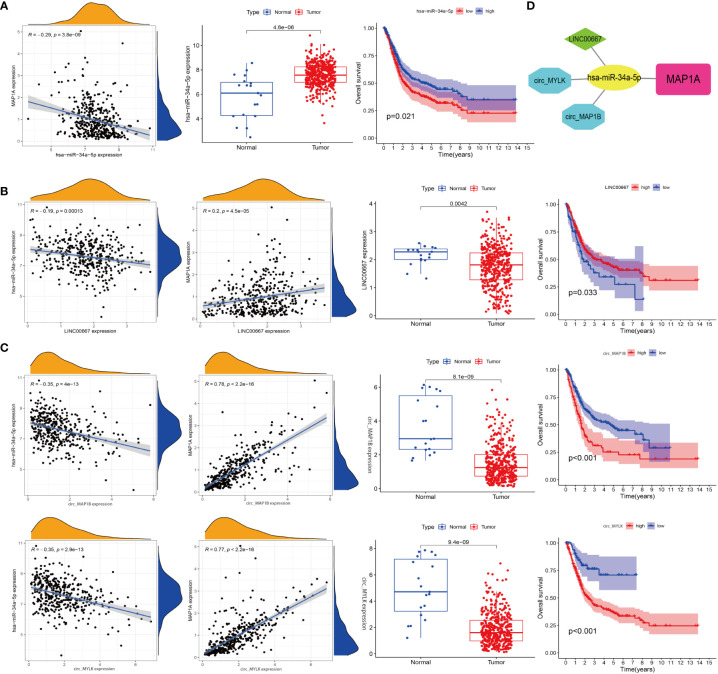
Construction of a ceRNA network. **(A)** Correlation analysis of miR-34a-5p with MAP1A, miR-34a-5p expression in bladder cancer tissues and paracancer tissues, and correlation analysis of miR-34a-5p expression with bladder cancer patients’ survival. **(B)** Analysis of the relationships between LINC0667, MAP1A, and miR-34a-5p expression, analysis of LINC0667 expression in bladder cancer and paracancer tissues, and correlation analysis of LINC0667 expression with bladder cancer patient survival. **(C)** Correlation analysis between circ_MAP1B and circ_MYLK with MAP1A and miR-34a-5p expression, analysis of circ_MAP1B and circ_MYLK expression in bladder cancer tissues and paracancer tissues, correlation analysis of circ_MAP1B and circ_MYLK expression with survival of bladder cancer patients. **(D)** Visualization map of ceRNA interaction network by cytoscape software.

Databases including microT, miRanda, miRmap, PITA, RNA22, PicTar, and Targetscan were used to predict the targeting miRNA of a MAP1A. A total of 69 miRNAs were predicted to bind MAP1A. TCGA-BLCA-miRNA expression data and clinical information were downloaded from the TCGA database. The Limma package in R (corFilter = 0.2, pvalueFilter = 0.001) was used to analyze differentially expressed miRNAs in bladder cancer. The survival package in R was applied to analyze bladder cancer survival-associated miRNA. Seven differentially expressed genes in bladder cancer tissues were found to correlate with survival in bladder cancer patients (P < 0.05). Additionally, a number of miRNAs (miR-182-5p, miR-15a-5p, miR-15b-5p, miR-16-5p, miR-671-5p, miR-185-5p, miR-24-3p, and miR-34a-5p) were identified that had a negative correlation with MAP1A expression. Then miR-34a-5p was finally selected according to the sponge-binging effect on MAP1A predicted in the 6 databases (microT, miRanda, miRmap, PITA, PicTar, Targetscan) (**Supplementary material**; **Table 3**). In addition, miR-34a-5p showed a negative correlation with MAP1A in bladder cancer tissues (P < 0.001, R = -0.29) and was related to bladder cancer **Supplementary material**, **Table 3** patient survival (P < 0.05) (**Figure 8A**).

Next, 139 interacted lncRNAs with miR-34a-5p were predicted through Starbase and TargetScan databases (**Supplementary material**, **Table 4**). Besides, 2696 interacted circRNAs were predicted using Targetscan, circbank, miRanda databanks (**Supplementary material**, **Table 5**). Limma package in R was used to identify genes correlated with miR-34a-5p and MAP1A in bladder cancer tissues. Two lncRNAs (LINC00667, CKMT2-AS1) and four circRNAs (hsa_circ_0050649, hsa_circ_0129524, hsa_circ_0067046, hsa_circ_0101667) that correlated with miR-34a-5p and MAP1A expression in bladder cancer tissues (negatively correlated with miR-34a-5p while positively correlated with MAP1A) were analyzed using the limma package in R. Both the limma and survival packages in R were applied to analyze the correlation between non-coding RNA expression and survival in bladder cancer patients, and 1 lncRNA and 3 circRNA (hsa_circ_0129524, hsa_circ_0067046, and circTNS1) (P < 0.001) were identified. Considering the negative correlation with miR-34a-5p and the positive correlation with MAP1A expression, LINC0667, circ_MYLK (hsa_circ_0067046), and circ_MAP1B (hsa_circ_0129524) were finally obtained (**Figure 8B**). Collectively, these results supported the idea that the MAP1A/miR-34a-5p/LINC0667/circ_MYLK/circ_MAP1B ceRNA network may play a role in the pathological processes of bladder cancer (**Figure 8C**).

## Discussion

In clinical bladder cancer patients, routine follow-up is required to monitor the disease progression after diagnosis and treatment. The routine follow-up visit generally includes a cystoscope, a urine cytological examination, and urography for the upper urinary tract. These frequent traumatic examinations not only affect patients’ quality of life but also increase their economic burden (32). Efforts are still underway to explore reliable biomarkers for the prognosis of bladder cancer. The present study aimed to identify biomarkers for early diagnosis and prognosis of bladder cancer clinically.

The TCGA transcriptome data and clinical data listed the differentially expressed mRNAs in bladder cancer at an early stage. Screening of microarray data of early-stage bladder cancer was also done with the GEO database, following WGCNA co-expression network analysis of both the TCGA-BLCA and GEO-BLCA databases. Finally, survival analysis of TCGA-BLCA data was conducted for the prediction of biomarkers related to the prognosis of bladder cancer. A total of 3 sets of overlap gene expression were obtained from TCGA-DEG, GEO-DEG, TCGA-WGCNA, GEO-WGCNA, and TCGA-Survival. After differential expression and survival analysis using the GEPIA and GETx databases, MAP1A was confirmed to have the strongest correlation with the prognosis of early-stage bladder cancer. High expression of MAP1A was noted in normal bladder tissues with a decreased expression in bladder cancer tissues. The same results were reflected *in vitro*, in both the bladder cancer cell lines SW780 and 5673 and the normal bladder epithelial cell SV-HUC-1.

The ceRNA network connects the function of protein-encoding mRNA with non-coding RNA, including microRNA, lncRNA, circRNA, and pseudogenes. All RNA with miRNA response element (MRE) can function as regulatory ceRNA. The mechanism of ceRNA involves competing endogenous genes sponge-binding target miRNA to regulate gene transcription. The activity of ceRNA is affected by various factors, including the length of ceRNA, the binding affinity of miRNA sponge, RNA secondary structure, and RNA-binding proteins. Abnormality in these factors may cause dysregulation of the ceRNA network and thus result in the development of diseases including cancer (33).

Therefore, in the present study, we predicted miRNA (miR-34a-5p) and ceRNAs (LINC0667/circ_MYLK/circ_MAP1B) involved in MAP1A expression regulation by constructing a MAP1A-miRNA-lncRNA/circRNA network. We retrieved the PubMed database and Web of Science database for current studies focused on the relationship between MAP1A and bladder cancer. A study by Yan et al. (34) reported that MAP1A is an autophagy-related gene associated with the prognosis of bladder cancer. In another study by Xie (35), MAP1S, a gene homologous to MAP1A, activates autophagy *via* regulation of the Beclin 1 dependent PIK3/AKT/mTOR pathway. The expression of MAP1S is positively correlated with the autophagy-related biomarker Bcl-2. Lv (36) demonstrated that miR-34a-5p induced cell autophagy through inhibition of Bcl-2 expression to activate the Beclin 1 signaling pathway. Meanwhile, miR-34a-5p is in negative correlation with Bcl-2 expression. ThePIK3/AKT/mTOR signaling pathway plays a critical role in the development and prognosis of bladder cancer (37). As a result, it was hypothesized that the miR-34a-5p mediated MAP1A ceRNA regulation network that promotes autophagy to induce bladder cancer develops through the PIK3-AKT-mTOR pathway. However, further experiments are required to identify the specific pathways involved in the development of bladder cancer.

Currently, TURBT in combination with immunochemical therapy is the main treatment for early-stage bladder cancer. Except for traditional treatment using BCG bladder irrigation, recently, immunotherapy targeting immune checkpoints CTLA-4 and PD-1/PD-L1 has been applied to treat early-stage bladder cancer, achieving satisfactory clinical efficacy (7, 38, 39). In order to predict whether MAP1A can be used to guide bladder cancer immunotherapy, we also analyzed the correlation of MAP1A with immune cells and immune checkpoints. According to Luo (40), the expression of MAP1A was closely correlated with the immunotherapeutic response of non-small cell lung cancer, and MAP1A is expected to serve as an immunotherapeutic target for non-small cell lung cancer (NSCLC). As shown in the results of our study on the correlation between MAP1A and immunotherapy, MAP1A is also possible to be a biomarker for monitoring immunotherapy response in bladder cancer.

The current research has certain limitations. For starters, *in vitro* and *in vivo* experiments failed to verify the mechanistic relationship between MAP1A and bladder carcinogenesis and development. Second, gene prediction for the prognosis of early-stage bladder cancer was focused only on the TCGA and GEO datasets. More data is still required for further research. Finally, further clinical investigation is aimed at confirming the interaction between MAP1A and bladder cancer stage and grading. *In vitro* and *in vivo* experiments will be performed in the future to explain the pathways of MAP1A in the development of bladder cancer. Clinical specimens of bladder cancer will be collected for verification.

## Conclusion

This study, using TCGA and GEO databases, predicted a potential biomarker correlation with the prognosis of bladder cancer by differential gene expression and weighted gene coexpression analysis. Further prediction uncovered its expression in pan-cancer, its correlation with immunocytes and immune checkpoints, and the ceRNA mechanism involved. Finally, we preliminarily tested MAP1A expression in bladder cancer cell lines. To our knowledge, this is the first time we predict and report the correlation of MAP1A with early diagnosis of bladder cancer, prognosis, immunotherapy, and the ceRNA network.

## Data availability statement

The datasets presented in this study can be found in online repositories. The names of the repository/repositories and accession number(s) can be found in the article/**Supplementary Material**.

## Author contributions

XL: investigation, methodology, data download, data analysis, writing- manuscript, editing and review. YQ: data collection. BZ: data collection. WX: data collection. YC: supervision. LM: guidance logicality, fund acquisition. All authors contributed to the article and approved the submitted version.

## Funding

This work supported by Shaanxi Province Key Research and Development Program (2020SF-192); the Xianyang City Social Development Project (2021ZDYF-SF-0046).

## Acknowledgments

We gratefully acknowledge the TCGA, GEO, GETx, UCSC Xena, GEPIA, TIMER database.

## Conflict of interest

The authors declare that the research was conducted in the absence of any commercial or financial relationships that could be construed as a potential conflict of interest.

## Publisher’s note

All claims expressed in this article are solely those of the authors and do not necessarily represent those of their affiliated organizations, or those of the publisher, the editors and the reviewers. Any product that may be evaluated in this article, or claim that may be made by its manufacturer, is not guaranteed or endorsed by the publisher.

## Abbreviations

MAP1A, Microtubule-associated Proteins 1A; TCGA, The Cancer Genome Atlas database; GEO, Gene Expression Omnibus; NCCN, National Comprehensive Cancer Network; AJCC, American Joint Committee on Cancer Staging; DEG, Differential expression gene; WGCNA, Weigth gene co-expression network analysis; BLCA, Bladder urothelial carcinoma; LC, Light chain; HPA, Human Protein Atlas database; KM, Kaplan-Meier; HR, Hazard ratio; CTLA-4, Cytotoxic T-lymphocyte-associated protein 4; PD-1, Programmed death 1; PD-L1, Programmed death ligand 1; ceRNA, Competing endogenous RNA; OS, Overall survival; DFS, Disease-free survival.

## References

[1] Bray F Ferlay J Soerjomataram I Siegel RL Torre LA Jemal A. Global cancer statistics 2018: GLOBOCAN estimates of incidence and mortality worldwide for 36 cancers in 185 countries. CA Cancer J Clin (2018) 68:394–424. doi: 10.3322/caac.21492 30207593

[2] Sung H Ferlay J Siegel RL Laversanne M Soerjomataram I Jemal A. Global cancer statistics 2020: GLOBOCAN estimates of incidence and mortality worldwide for 36 cancers in 185 countries. CA Cancer J Clin (2021) 71:209–49. doi: 10.3322/caac.21660 33538338

[3] Thomas W. Flaig. NCCN guidelines version 4.2020 bladder cancer. NCCN Clin Pract Guidel Oncol (NCCN Guidel (2020) 6:224–43. doi: 10.6004/jnccn.2020.0011

[4] Guney S Guney N Canogullari Z Ergenekon E. TA T1 low and intermediate transitional cell carcinoma of the bladder: Recurrence rates and the timing of check cystoscopies within the first year. Urol Int (2008) 80:124–8. doi: 10.1159/000112600 18362479

[5] Harshman LC Preston MA Bellmunt J Beard C. Diagnosis of bladder carcinoma: A clinician’s perspective. Surg Pathol Clin (2015) 8:677–85. doi: 10.1016/j.path.2015.07.004 26612221

[6] Chang SS Boorjian SA Chou R Clark PE Daneshmand S Konety BR. Diagnosis and treatment of non-muscle invasive bladder cancer: AUA/SUO guideline. J Urol (2016) 196:1021–9. doi: 10.1016/j.juro.2016.06.049 27317986

[7] Miller KD Nogueira L Mariotto AB Rowland JH Yabroff KR Alfano CM. Cancer treatment and survivorship statistics, 2019. CA Cancer J Clin (2019) 69:363–85. doi: 10.3322/caac.21565 31184787

[8] Siegel RL Miller KD Fuchs HE Jemal A. Cancer statistics, 2021. CA Cancer J Clin (2021) 71:7–33. doi: 10.3322/caac.21654 33433946

[9] Mei X Sweatt AJ Hammarback JA. Microtubule-associated protein 1 subunit expression in primary cultures of rat brain. Brain Res Bull (2000) 53:801–6. doi: 10.1016/S0361-9230(00)00416-0 11179846

[10] Fink JK Jones SM Esposito C Wilkowski J. Human microtubule-associated protein 1a (MAP1A) gene: Genomic organization, cDNA sequence, and developmental- and tissue-specific expression. Genomics (1996) 35:577–85. doi: 10.1006/geno.1996.0400 8812494

[11] Halpain S Dehmelt L. The MAP1 family of microtubule-associated proteins. Genome Biol (2006) 7:1–7. doi: 10.1186/gb-2006-7-6-224 PMC1779536 16938900

[12] Lajoie-Mazenc I Tovar D Penary M Lortal B Allart S Favard C. MAP1A light chain-2 interacts with GTP-RhoB to control epidermal growth factor (EGF)-dependent EGF receptor signaling. J Biol Chem (2008) 283:4155–64. doi: 10.1074/jbc.M709639200 18056259

[13] Wettergren EE Gussing F Quintino L Lundberg C. Novel disease-specific promoters for use in gene therapy for parkinson’s disease. Neurosci Lett (2012) 530:29–34. doi: 10.1016/j.neulet.2012.09.059 23063686

[14] Meyer MA. Highly expressed genes in human high grade gliomas: Immunohistochemical analysis of data from the human protein atlas. Neurol Int (2014) 6:29–31. doi: 10.4081/ni.2014.5348 PMC4077206 24987500

[15] Kang HJ Kim KT Yoo KH Park Y Kim JW Kim SW. Genetic markers for later remission in response to early improvement of antidepressants. Int J Mol Sci (2020) 21:1–18. doi: 10.3390/ijms21144884 PMC7402334 32664413

[16] Fernandez J Portilho DM Danckaert A Munier S Becker A Roux P. Microtubule-associated proteins 1 (MAP1) promote human immunodeficiency virus type I (HIV-1) intracytoplasmic routing to the nucleus. J Biol Chem (2015) 290:4631–46. doi: 10.1074/jbc.M114.613133 PMC4335204 25505242

[17] Jiang X Zhong W Huang H He H Jiang F Chen Y. Autophagy defects suggested by low levels of autophagy activator MAP1S and high levels of autophagy inhibitor LRPPRC predict poor prognosis of prostate cancer patients. Mol Carcinog (2015) 54:1194–204. doi: 10.1002/mc.22193 PMC4941638 25043940

[18] Lindgren D Frigyesi A Gudjonsson S Sjödahl G Hallden C Chebil G. Combined gene expression and genomic profiling define two intrinsic molecular subtypes of urothelial carcinoma and gene signatures for molecular grading and outcome. Cancer Res (2010) 70:3463–72. doi: 10.1158/0008-5472.CAN-09-4213 20406976

[19] Cline MS Craft B Swatloski T Goldman M Ma S Haussler D. Exploring TCGA pan-cancer data at the UCSC cancer genomics browser. Sci Rep (2013) 3:2652. doi: 10.1038/srep02652 24084870 PMC3788369

[20] Robinson MD McCarthy DJ Smyth GK. edgeR: A bioconductor package for differential expression analysis of digital gene expression data. Bioinformatics (2010) 26:139–40. doi: 10.1093/bioinformatics/btp616 PMC2796818 19910308

[21] Hokamp K. Perl One-liners: Bridging the gap between Large data sets and analysis tools. Methods Mol Biol (2015) 1326:1326. doi: 10.1007/978-1-4939-2839-2_15 26498621

[22] Ritchie ME Phipson B Wu D Hu Y Law CW Shi W. Limma powers differential expression analyses for RNA-sequencing and microarray studies. Nucleic Acids Res (2015) 43:e47. doi: 10.1093/nar/gkv007 25605792 PMC4402510

[23] In J Lee DK. Survival analysis: part II – applied clinical data analysis. Korean J Anesth (2019) 72:441–57. doi: 10.4097/kja.19183 PMC6781220 31096731

[24] Goel MK Khanna P Kishore J. Understanding survival analysis: Kaplan-Meier estimate. Int J Ayurveda Res (2010) 1:274–8. doi: 10.4103/0974-7788.76794 PMC3059453 21455458

[25] George B Seals S Aban I. Survival analysis and regression models. J Nucl Cardiol (2014) 21:686–94. doi: 10.1007/s12350-014-9908-2 PMC4111957 24810431

[26] Langfelder P Horvath S. WGCNA: An r package for weighted correlation network analysis. BMC Bioinf (2008) 9 :559. doi: 10.1186/1471-2105-9-559 PMC2631488 19114008

[27] Li T Fan J Wang B Traugh N Chen Q Liu JS. TIMER: A web server for comprehensive analysis of tumor-infiltrating immune cells. Cancer Res (2017) 77:e108–10. doi: 10.1158/0008-5472.CAN-17-0307 PMC6042652 29092952

[28] Li JH Liu S Zhou H Qu LH Yang JH. StarBase v2.0: Decoding miRNA-ceRNA, miRNA-ncRNA and protein-RNA interaction networks from large-scale CLIP-seq data. Nucleic Acids Res (2014) 42:92–7. doi: 10.1093/nar/gkt1248 PMC3964941 24297251

[29] Liu S Xie X Lei H Zou B Xie L. Identification of key circRNAs/lncRNAs/miRNAs/mRNAs and pathways in preeclampsia using bioinformatics analysis. Med Sci Monit Int Med J Exp Clin Res (2019) 25:1679–93. doi: 10.12659/MSM.912801 PMC6413561 30833538

[30] Maragkakis M Reczko M Simossis VA Alexiou P Papadopoulos GL Dalamagas T. DIANA-microT web server: elucidating microRNA functions through target prediction. Nucleic Acids Res (2009) 37:W273–6. doi: 10.1093/nar/gkp292 PMC2703977 19406924

[31] Liu M Wang Q Shen J Yang BB Ding X. Circbank: a comprehensive database for circRNA with standard nomenclature. RNA Biol (2019) 16:899–905. doi: 10.1080/15476286.2019.1600395 31023147 PMC6546381

[32] Babjuk M Burger M Compérat EM Gontero P Mostafid AH Palou J. European Association of urology guidelines on non-muscle-invasive bladder cancer (TaT1 and carcinoma in situ) - 2019 update. Eur Urol (2019) 76:639–57. doi: 10.1016/j.eururo.2019.08.016 31443960

[33] Qi X Zhang DH Wu N Xiao JH Wang X Ma W. ceRNA in cancer: Possible functions and clinical implications. J Med Genet (2015) 52:710–8. doi: 10.1136/jmedgenet-2015-103334 26358722

[34] Yan X Wu HH Chen Z Du GW Bai XJ Tuoheti K. Construction and validation of an autophagy-related prognostic signature and a nomogram for bladder cancer. Front Oncol (2021) 11:632387. doi: 10.3389/fonc.2021.632387 34221960 PMC8252967

[35] Xie R Nguyen S McKeehan K Wang F McKeehan WL Liu L. Microtubule-associated protein 1S (MAP1S) bridges autophagic components with microtubules and mitochondria to affect autophagosomal biogenesis and degradation. J Biol Chem (2011) 286:10367–77. doi: 10.1074/jbc.M110.206532 PMC3060490 21262964

[36] Lv X Wang K Tang W Yu L Cao H Chi W. miR - 34a - 5p was involved in chronic intermittent hypoxia - induced autophagy of human coronary artery endothelial cells *via* bcl - 2 / beclin 1 signal transduction pathway. (2019) 120: 18871–82. doi: 10.1002/jcb.29207 31218746

[37] Korkolopoulou P Levidou G Trigka EA Prekete N Karlou M Thymara I. A comprehensive immunohistochemical and molecular approach to the PI3K/AKT/mTOR (phosphoinositide 3-kinase/v-akt murine thymoma viral oncogene/mammalian target of rapamycin) pathway in bladder urothelial carcinoma. BJU Int (2012) 110:E1237–48. doi: 10.1111/j.1464-410X.2012.11569.x 23107319

[38] Zhou TC Sankin AI Porcelli SA Perlin DS Schoenberg MP Zang X. A review of the PD-1/PD-L1 checkpoint in bladder cancer: From mediator of immune escape to target for treatment. Urol Oncol Semin Orig Investig (2017) 35:14–20. doi: 10.1016/j.urolonc.2016.10.004 27816403

[39] Powles T Necchi A Rosen G Hariharan S Apolo AB. Anti–programmed cell death 1/Ligand 1 (PD-1/PD-L1) antibodies for the treatment of urothelial carcinoma: State of the art and future development. Clin Genitourin Cancer (2018) 16:117–29. doi: 10.1016/j.clgc.2017.11.002 PMC5878995 29325739

[40] Luo J Hu Q Gou M Liu X Qin Y Zhu J. Expression of microtubule-associated proteins in relation to prognosis and efficacy of immunotherapy in non-small cell lung cancer. Front Oncol (2021) 11:680402. doi: 10.3389/fonc.2021.680402 34660263 PMC8517487

